# A retrospective study of the aetiology of increased mean platelet volume in dogs presented to a small animal teaching hospital in Greece

**DOI:** 10.1002/vro2.70000

**Published:** 2024-12-03

**Authors:** Ioannis L. Oikonomidis, Harris Antoniadis, Afroditi Papathanasiou, Theodora K. Tsouloufi, Timokleia Kousi, Maria Rafaella Kalafati, Maria Kritsepi‐Konstantinou

**Affiliations:** ^1^ School of Veterinary Medicine Faculty of Health Sciences Aristotle University of Thessaloniki Thessaloniki Greece; ^2^ Department of Veterinary Anatomy, Physiology and Pathology Institute of Infection Veterinary and Ecological Sciences University of Liverpool, Wirral Neston UK

**Keywords:** canine, platelet activation, platelet size, thrombocytopenia, thrombopoiesis

## Abstract

**Background:**

The aetiology of increased mean platelet volume in dogs is currently uncertain. Our aim was to investigate the aetiology of increased mean platelet volume in dogs with and without thrombocytopenia.

**Methods:**

The database of a teaching hospital was retrospectively searched for dogs with increased mean platelet volume (>14.4 fL) over a 3‐year period. Complete blood counts were performed with an Advia 120 analyser. Cases with incomplete medical records or belonging to breeds known to be associated with macrothrombocytopenia were excluded.

**Results:**

Sixty‐six dogs were included, with Group 1 consisting of non‐thrombocytopenic dogs (49/66, 74.2%) and Group 2 consisting of thrombocytopenic dogs (17/66, 25.8%). Diagnoses significantly differed between the two groups (*p* = 0.003). In Group 1, inflammatory/infectious diseases (69.4%) were the most common cause, followed by neoplastic diseases (8.2%) and diabetes mellitus (6.1%). Dogs in Group 2 were diagnosed with either inflammatory/infectious diseases (50.0%) or neoplastic diseases (50.0%). The small sample size‐ and a potential delayed haematological analysis of some of the blood samples, could have artifactually affected the mean platelet volume.

**Conclusions:**

In dogs with thrombocytopenia and increased mean platelet volume, inflammatory/infectious or neoplastic diseases should be considered. In non‐thrombocytopenic dogs, increased mean platelet volume is primarily associated with inflammatory/infectious diseases, with neoplasia and diabetes mellitus being infrequent causes.

## INTRODUCTION

The mean platelet volume (MPV) is a measurement of platelet size provided by automated haematology analysers. In both humans and dogs, an inverse relationship between the platelet count and MPV has been reported, which aims to preserve a constant platelet mass.^1^, ^2^ Platelet morphology and function depend on factors affecting megakaryopoiesis/thrombopoiesis and platelet circulation, with the former likely having the highest impact on the size and function of platelets.^3^ An increased MPV indicates active/accelerated thrombopoiesis and has been associated with adequate to increased numbers of megakaryocytes on bone marrow cytology in people and dogs.^4^, ^5^, ^6^ It has been suggested that MPV is expected to be increased in human patients with thrombocytopenia caused by increased platelet destruction, consumption or loss.^7^, ^8^ A few studies have evaluated the use of MPV as a discriminatory marker between dogs with thrombocytopenia due to decreased platelet production or increased platelet destruction, consumption or loss. While one study associated immune thrombocytopenia with increased MPV,^9^ another study did not confirm this finding.^10^ In a study of thrombocytopenic dogs, MPV was not found useful in differentiating the underlying mechanism of thrombocytopenia.^1^

Several hormones and cytokines, most importantly thrombopoietin, granulocyte‐macrophage colony‐stimulating factor, interleukin (IL)‐1, IL‐6 and tumour necrosis factor (TNF)‐α, play significant roles in megakaryopoiesis and thrombopoiesis.^11^ Consequently, changes in MPV can reflect both prothrombotic and proinflammatory conditions. Indeed, in human medicine, there is growing evidence supporting the clinical usefulness of MPV in non‐thrombocytopenic individuals, particularly in inflammatory diseases and conditions associated with an increased risk of thrombosis.^2^, ^12^, ^13^

Even though MPV is readily available as a part of the complete blood count (CBC) and despite its potential utility, it is often not evaluated in dogs, especially in those without thrombocytopenia. This is primarily attributed to the limited available literature. The use of MPV as a potential diagnostic or prognostic marker, independent of the presence of thrombocytopenia, has been scarcely studied in dogs.^14^, ^15^, ^16^, ^17^ The MPV was found to be significantly higher in dogs with parvoviral enteritis,^17^ pancreatitis^15^ and septic peritonitis,^14^ when compared to healthy, non‐thrombocytopenic dogs.

The objective of our study was to investigate the aetiology of increased MPV in dogs with and without thrombocytopenia. We hypothesised that the aetiology of increased MPV might differ between thrombocytopenic and non‐thrombocytopenic dogs, and increased MPV would primarily be associated with inflammatory/infectious diseases in non‐thrombocytopenic dogs.

## MATERIALS AND METHODS

An ethical approval was not required because this was a retrospective study. The electronic database of the Diagnostic Laboratory, School of Veterinary Medicine, Aristotle University of Thessaloniki, Greece was retrospectively searched for dogs with increased MPV over a period of 3 years (January 2015 to December 2017). Increased MPV was defined as greater than 14.4 fL‐ and the reference interval for platelet count was 173,000–487,000/µL, according to previously published species‐ and analyser‐specific reference intervals.^18^ In the small animal teaching hospital where the study was performed, jugular venipuncture is typically used for blood collection into K_3_EDTA tubes (Eurotubo)‐ and the blood samples are immediately submitted to the diagnostic laboratory for analysis. All CBCs were performed using the Siemens Advia 120 (Siemens Healthcare Diagnostics) and the relevant species‐specific software. In our laboratory, the CBCs were typically completed within 30–60 minutes of blood collection and blood smears were evaluated by intern veterinarians for all dogs with thrombocytopenia.

The medical records of the identified cases were reviewed and cases with incomplete medical records were excluded from the study. The retrieved data included (i) age; (ii) breed; (iii) sex; (iv) CBC results including the findings from the blood film examination and (v) diagnosis. The diagnoses were categorised into the following groups, as previously reported^1^: neoplasia, inflammatory/infectious diseases, immune‐mediated diseases, allergic diseases, endocrine diseases, degenerative diseases, developmental conditions or miscellaneous. Dogs belonging to Cavalier King Charles spaniel, Norfolk terrier, Cairn terrier and Akita breeds were excluded from the study, because macrothrombocytopenia is considered a normal finding in these breeds.^19^, ^20^, ^21^ Additionally, healthy dogs were excluded. Thrombocytopenic dogs with platelet clumping on the examined blood smear were also excluded.

The distribution of data was assessed using the Shapiro–Wilk test. The chi‐square test was used to examine the association between categorical variables. The Wilcoxon rank sum test was used to compare numerical variables between two groups. Spearman's correlation coefficient was used to examine the correlation between two variables. All statistical analyses were performed using the statistical language R, version 4.3.0 (R Foundation for Statistical Computing). For all tests applied, a *p*‐value less than 0.05 was considered significant.

## RESULTS

**FIGURE 1:**
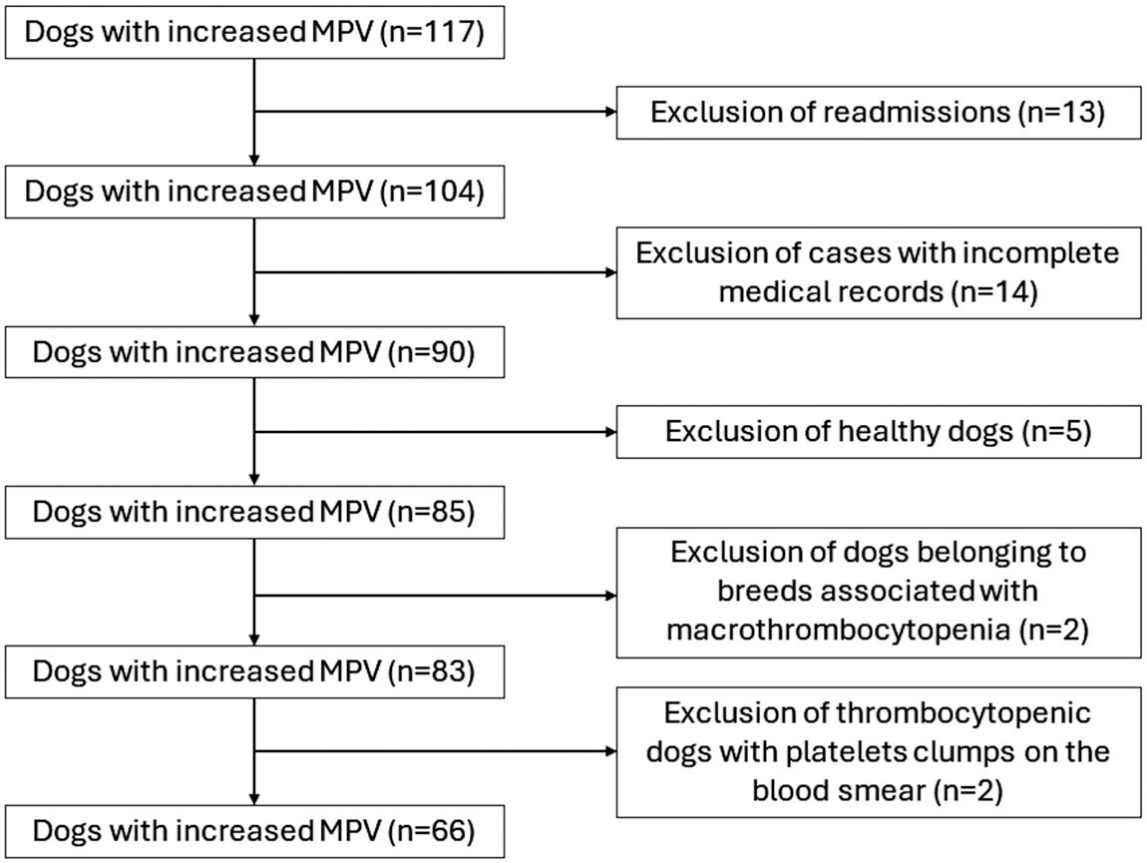
Detailed flowchart of case exclusion criteria.

In total, we identified 117 cases with increased MPV. Thirteen cases were readmissions and were excluded. Among them, 14 cases were excluded due to incomplete medical records. Additionally, five healthy dogs, one Akita and one Cavalier King Charles spaniel dog were excluded based on our predefined exclusion criteria. Another 17 dogs with thrombocytopenia were excluded because of the presence of platelet clumping on the examined blood smear. Ultimately, 66 dogs were included for further analysis (Figure 1). None of the dogs were receiving medical treatment for the preceding 2 weeks. Out of the 66 dogs, 39 (59.1%) had platelet counts within the reference interval, 17 (25.8%) had thrombocytopenia and 10 (15.1%) had thrombocytosis. The dogs were allocated into two groups: Group 1 consisting of dogs without thrombocytopenia and Group 2 consisting of dogs with thrombocytopenia.

The descriptive statistics for age, sex and breeds for each of the two groups are presented in Table 1. The median age between the two groups was not statistically significant (*p* = 0.130). The median MPV was significantly lower in Group 1 [16.5 fL (14.5–22.8 fL)] as compared to Group 2 [19.6 fL (15.4–22.3 fL)] (*p* = 0.001). The median platelet count was 303,000/µL (178,000–1,351,000/µL) in Group 1 and 66,000/µL (31,000–146,000/µL) in Group 2. This difference was statistically significant (*p* < 0.001). A statistically significant negative correlation was found between the MPV and platelet count in our population (rho = −0.318, *p* = 0.009).

**TABLE 1:** Descriptive statistics for age, sex and breed of the non‐thrombocytopenic (Group 1) and thrombocytopenic dogs (Group 2).

Variable	Group 1 (*n* = 49)	Group 2 (*n* = 17)
Age, median (range) in years	6.0 (0.1–15.0)	7.0 (2.0–16.0)
Sex	26 male, 23 female	10 male, 7 female
Breed	One each of beagle, boxer, chihuahua, dobermann, French bulldog, German shepherd dog, Maltese, Pekingese, pit bull, Yorkshire terrier. Two each of cocker spaniel, English setter, Greek hound and poodle; mixed‐breed (*n* = 26), Rottweiler (*n* = 5)	One each cocker spaniel, German shepherd dog, Pekingese, pointer and poodle; mixed‐breed (*n* = 10), pit bull (*n* = 2)

A statistically significant difference in the distribution of diagnoses between Groups 1 and 2 was noted (*χ* ^2^ = 18.189, df = 5, *p* = 0.003). In non‐thrombocytopenic dogs, the most common cause of increased MPV was inflammatory/infectious diseases (69.4%), followed by neoplasia (8.2%) and diabetes mellitus (6.1%) (Tables 2 and 3). Thrombocytopenic dogs with increased MPV were equally diagnosed with inflammatory/infectious or neoplastic diseases (Table 2).

**TABLE 2:** Disease category of 66 dogs with increased mean platelet volume.

Disease group	Group 1 (*n* = 49)	Group 2 (*n* = 17)
Degenerative	2 (4.1%)	0
Endocrine	3 (6.1%)	0
Inflammatory/infectious	34 (69.4%)	9 (50.0%)^a^
Miscellaneous	6 (12.2%)	0
Neoplasia	4 (8.2%)	9 (50.0%)^a^

*Note*: The non‐thrombocytopenic dogs were allocated into Group 1‐ and thrombocytopenic dogs into Group 2.

^a^ One dog had both neoplasia (lymphoma) and an inflammatory/infectious disease (dirofilariasis).

**TABLE 3:** Diagnoses for 66 dogs with increased mean platelet volume.

Disease	Group 1 (*n* = 49)^a^	Group 2 (*n* = 17)
Acute gastroenteritis	9 (18.4%)	0
Bladder sphincter atony	1 (2.0%)	0
Chronic hepatitis	0	1 (5.9%)
Degenerative mitral valve disease	1 (2.0%)	0
Diabetes mellitus	3 (6.1%)	0
Dirofilariasis	3 (6.1%)	1 (5.9%)
Ehrlichiosis	1 (2.0%)	1 (5.9%)
External trauma	1 (2.0%)	0
Hernia	3 (6.1%)	0
Idiopathic epilepsy	1 (2.0%)	0
Intervertebral disc disease	1 (2.0%)	0
Leishmaniosis	9 (18.4%)	1 (5.9%)
Neoplasia^b^	4 (8.2%)	8 (47.1%)
Multiple^c^	4 (8.2%)	3 (17.6%)
Pancreatitis	0	1 (5.9%)
Periodontitis	3 (6.1%)	0
Septic peritonitis	1 (2.0%)	0
Skin abscess	1 (2.0%)	0
Tracheobronchitis	1 (2.0%)	1 (5.9%)
Urinary tract infection	2 (4.1%)	0

*Note*: The non‐thrombocytopenic dogs were allocated into Group 1‐ and thrombocytopenic dogs into Group 2.

^a^ Group 1 included 39 dogs with platelet count within the reference interval and 10 dogs with thrombocytosis. The diagnoses of these 10 dogs were diabetes mellitus (*n* = 3), leishmaniosis (*n* = 2), parasitic enteritis (*n* = 2), with one each of periodontitis, urinary tract infection and bone fracture, and perineal hernia.

^b^ Group 1 included one each of hepatic sarcoma, lipoma, pharyngeal transitional cell carcinoma and splenic haemangiosarcoma (*n* = 4). Group 2 included gastric sarcoma (*n* = 1), hepatocellular neoplasm (*n* = 2), hepatosplenic lymphoma (*n* = 2), multicentric lymphoma (*n* = 1), ocular lymphoma (*n* = 1) and splenic haemangiosarcoma (*n* = 1).

^c^ Group 1 included one each of urinary tract infection and bone fracture; leishmaniosis and external trauma; urinary tract infection, urolithiasis and uroperitoneum; and idiopathic colitis and otitis due to *Malassezia* spp. (*n* = 4). Group 2 included one each of leishmaniosis and ehrlichiosis, leishmaniosis and dirofilariasis, also dirofilariasis with lymphoma.

## DISCUSSION

According to the results of our study, the incidence of diseases associated with increased MPV differs between thrombocytopenic and non‐thrombocytopenic dogs. In thrombocytopenic dogs, increased MPV was associated with both inflammatory/infectious and neoplastic diseases at equal frequencies. In contrast, in dogs without thrombocytopenia, increased MPV was predominantly reported in association with inflammatory/infectious diseases, with a small percentage of dogs having neoplasia or diabetes mellitus.

As hypothesised, inflammatory/infectious diseases were the most common conditions associated with increased MPV in non‐thrombocytopenic dogs, accounting for almost 70% of the cases. In inflammatory conditions, the release of proinflammatory cytokines, notably IL‐6, leads to increased production of thrombopoietin.^11^, ^22^ The increased production of thrombopoietin, IL‐6 and other proinflammatory cytokines leads to increased production of large immature platelets and increased platelet numbers per unit volume.^11^, ^22^, ^23^, ^24^ According to human studies, at the same time, large platelets, which are believed to be more active, rapidly migrate to the site of inflammation where they are consumed.^25^, ^26^ This might lead to a decrease in MPV during ongoing inflammation, as observed in human patients with inflammatory conditions.^25^, ^26^ Assuming that this is also likely to be true in dogs, it is important to emphasise that a normal or even decreased MPV does not exclude the presence of inflammation. The most common diagnoses in this group of dogs were leishmaniosis (18.4%) and acute gastroenteritis (18.4%). Therefore, acute gastroenteritis should be high on the list of differentials in dogs with increased MPV and absence of thrombocytopenia, while in endemic areas like the one where this study was performed, leishmaniosis appears to be a very important differential diagnosis as well. However, it should be noted that the prevalence of some infectious diseases, as mentioned for leishmaniosis, directly depends on the geographical location.

Inflammatory/infectious diseases were also diagnosed in 50% of the thrombocytopenic dogs included in our study. Diagnoses included leishmaniosis, ehrlichiosis, dirofilariasis, pancreatitis, chronic hepatitis and tracheobronchitis. What was previously given for non‐thrombocytopenic dogs, with increased MPV diagnosed with inflammatory/infectious diseases, also applies to thrombocytopenic dogs. Additionally, previous studies have demonstrated the association of leishmaniosis, ehrlichiosis and sporadically a variety of other inflammatory conditions with immune thrombocytopenia, as evidenced by the presence of anti‐platelet antibodies.^10^, ^27^, ^28^, ^29^, ^30^, ^31^, ^32^ This could further explain the observed increase in MPV, as previously noted.^9^

Among the non‐thrombocytopenic dogs with increased MPV, only 8.2% were diagnosed with neoplasia, which is substantially lower compared to the 50% incidence observed in thrombocytopenic dogs. Several neoplasms have been associated with alterations in platelet indices, such as MPV and platelet distribution width in human patients.^33^, ^34^ It is challenging to determine the underlying mechanism of increased MPV as well as thrombocytopenia in cases with neoplasia. Lymphoma, hepatic neoplasia and splenic haemangiosarcoma constituted 75% of neoplastic cases. Lymphoma has been associated with immune thrombocytopenia,^10^ which can be associated with increased MPV.^9^ Increased platelet consumption or loss may be the primary cause of thrombocytopenia and increased MPV in cases of splenic haemangiosarcoma and hepatic neoplasia, while the close association between inflammation and neoplasia may also play a role and contribute to increased MPV, especially in non‐thrombocytopenic dogs with neoplasia.^35^ Our findings suggest that neoplasia, while a common cause of thrombocytopenia with increased MPV, appears to be an uncommon cause of increased MPV in non‐thrombocytopenic dogs. However, given the small sample size of our study, this finding should be confirmed using a larger canine population.

It is noteworthy that three (6.1%) non‐thrombocytopenic dogs with increased MPV were diagnosed with diabetes mellitus. All three dogs had thrombocytosis. In one study, thrombocytosis was a common finding in diabetic dogs.^36^ According to the results of the same study, median MPV was not significantly different in diabetic dogs compared to healthy individuals, although it was numerically higher.^36^ However, some of the diabetic dogs included in that study had increased MPV.^36^ Therefore, diabetes mellitus should also be considered as a possible differential diagnosis in dogs with increased MPV, especially in the presence of thrombocytosis.

Our study has two significant limitations that should be acknowledged. First, the small number of cases included in both the thrombocytopenic and non‐thrombocytopenic groups may limit the robustness of the conclusions drawn. Nevertheless, this study serves as an initial exploration of the utility of MPV as a diagnostic marker in dogs without thrombocytopenia. Another limitation to consider is related to the retrospective nature of our study. Although CBCs are typically completed within 30–60 minutes of blood collection in the laboratory where the study was performed, it is important to note the potential for a delay in the haematological analysis of certain blood samples. Such delays could potentially lead to artefactual changes in MPV.

Under the conditions of our study, inflammatory/infectious diseases were by far the most common cause of increased MPV in non‐thrombocytopenic dogs and were also a common cause of increased MPV in thrombocytopenic dogs. Although neoplasia was a common cause of thrombocytopenia with increased MPV, it appears to be an uncommon cause of increased MPV in non‐thrombocytopenic dogs. Diabetes mellitus should also be considered as a possible, but infrequent, cause of increased MPV, especially in the presence of thrombocytosis. More studies with larger sample size and prospective design are required to further explore the utility of MPV in clinical practice.

## AUTHOR CONTRIBUTIONS

Ioannis L. Oikonomidis: conceived the idea; did the statistical analysis and wrote the manuscript. Harris Antoniadis; Afroditi Papathanasiou; Timokleia Kousi; Maria Rafaella Kalafati: collected the data and proofread the manuscript. Theodora K. Tsouloufi: conceived the idea and proofread the manuscript. Maria Kritsepi‐Konstantinou: supervised the collection of data and proofread the manuscript.

## CONFLICTS OF INTEREST STATEMENT

The authors declare they have no conflicts of interest.

## FUNDING INFORMATION

The authors received no specific funding for this work.

## ETHICS STATEMENT

An ethical approval was not required because this was a retrospective study.

## Data Availability

Data available on request from the authors.
